# Maggot extracts chemo-prevent inflammation and tumorigenesis accompanied by changes in the intestinal microbiome and metabolome in AOM/DSS-induced mice

**DOI:** 10.3389/fmicb.2023.1143463

**Published:** 2023-05-02

**Authors:** Xun Tang, Lei Wang, Daojuan Wang, Yi Zhang, Tingyu Wang, Zhengquan Zhu, Yajing Weng, Gaojian Tao, Qin Wang, Li Tang, Feng Yan, Yong Wang

**Affiliations:** ^1^State Key Laboratory of Analytical Chemistry for Life Science and Jiangsu Key Laboratory of Molecular Medicine, The Affiliated Nanjing Drum Tower Hospital, Medical School of Nanjing University, Nanjing, China; ^2^Department of Clinical Laboratory, The Affiliated Cancer Hospital of Nanjing Medical University and Jiangsu Cancer Hospital and Jiangsu Institute of Cancer Research, Nanjing, China; ^3^Department of Clinical Laboratory, The Affiliated Hospital of Integrated Traditional Chinese and Western Medicine, Nanjing University of Chinese Medicine, Jiangsu Province Academy of Traditional Chinese Medicine, Nanjing, China; ^4^Department of Pathology, The Affiliated Cancer Hospital of Nanjing Medical University and Jiangsu Cancer Hospital and Jiangsu Institute of Cancer Research, Nanjing, China; ^5^Nanjing University (Suzhou) High-Tech Institute, Nanjing University, Suzhou, China

**Keywords:** maggot extract (ME), inflammation, colitis-associated colon cancer (CAC), intestinal microbiota, metabolome

## Abstract

Inflammatory responses and intestinal microbiome play a crucial role in the progression of colitis-associated carcinoma (CAC). The traditional Chinese medicine maggot has been widely known owing to its clinical application and anti-inflammatory function. In this study, we investigated the preventive effects of maggot extract (ME) by intragastric administration prior to azoxymethane (AOM) and dextran sulfate sodium (DSS)-induced CAC in mice. The results showed that ME had superior advantages in ameliorating disease activity index score and inflammatory phenotype, in comparison with the AOM/DSS group. The number and size of polypoid colonic tumors were decreased after pre-administration of ME. In addition, ME was found to reverse the downregulation of tight junction proteins (zonula occluden-1 and occluding) while suppressing the levels of inflammatory factors (IL-1β and IL-6) in models. Moreover, Toll-like receptor 4 (TLR4) mediated intracellular nuclear factor-κB (NF-κB)-containing signaling cascades, including inducible nitric oxide synthase and cyclooxygenase-2, and exhibited decreasing expression in the mice model after ME pre-administration. 16s rRNA analysis and untargeted-metabolomics profiling of fecal samples inferred that ME revealed ideal prevention of intestinal dysbiosis in CAC mice, accompanied by and correlated with alterations in the composition of metabolites. Overall, ME pre-administration might be a chemo-preventive candidate in the initiation and development of CAC.

## 1. Introduction

Colorectal cancer (CRC) is the third most common malignancy and the second mortality of cancer death globally (Siegel et al., 2020). Inflammatory factors, such as bowel disease (IBD), play an etiologic part in CRC, predisposing patients to a high risk of morbidity and cancerization (Blackman et al., 2021). The epidemiological investigation has indicated that IBD cancer accounts for only 1–2% of CRC, but it is the main cause of death for IBD patients that is often recurrent (Li et al., 2019). Indeed, such a causal link between chronic inflammation and colitis-associated carcinoma (CAC) has served to be confirmed. A tumor microenvironment containing the immune cells that secrete proinflammatory and anti-inflammatory factors and release reactive oxygen and nitrogen species have been suspected to promote tumor initiation and progression (Chen et al., 2021; Overacre-Delgoffe et al., 2021). Another direct example of the transformation of inflammatory cancer was supplied by mucosa-associated lymphoid tissue (MALT) lymphoma, which was caused by chronic infection from persistent activation of B cells to genetic arrangement leading to carcinogenesis eventually (Thieblemont et al., 2014). Anti-inflammatory treatment is very important and effective, so chemo-prevention strategies are necessary. Nowadays, food-origin and herb-origin products with diverse functions are emerged as novel chemo-prevention agents and are used in clinical medicine owing to their anti-inflammatory effects and safe benefits (Chung et al., 2018; Fong et al., 2020; Chen et al., 2021; Iqbal et al., 2021; Sameni et al., 2021). In addition, newer precision medicine, such as aspirin (acetylsalicylic acid), is placed with great hopes to get better control of the potential risks of cancerization (Gilligan et al., 2019; Hua et al., 2019).

The gut microbiome (GMB) is very large, and its interaction with the human body is highly complex. Studies had shown that the balance of GMB played a crucial role in intestinal immunity and host health, and changes in GMB might lead to a variety of metabolomics through their metabolites (Stutz et al., 2022). On the other hand, dysbiosis of the microbial population may promote mucosal injury by means of driving gut inflammation (Bajic et al., 2020; Dooyema et al., 2022). Accumulating evidence suggests that intestinal microorganism disorder in CAC patients induces an abnormal immune response, destroys intestinal homeostasis, and eventually leads to the loss of intestinal mucosal barrier integrity (Tilg et al., 2020). Pathogenic or probiotic bacterial infection is a key part of the triggering of IBD cancerization. For instance, *Akkermansia muciniphila*, a type of probiotic, can repair the gut barrier and blunt CAC by modulation of immune cells (Wang et al., 2022a). A link between dysbacteriosis and CAC was validated. It is, thus, urgent to find approaches to reverse intestinal flora and barrier function as a notable strategy in the prevention of CAC (Wang et al., 2020a; Chang et al., 2022).

The Chinese medicine maggot is the larva of *Chrysomya megacephala* (Fabricius, named “larvae of *Lucilia sericata”* in Latin) and its relatives, belonging to the Calliphoridae family. Maggot was widely used in traditional prescriptions and such classic works as “Compendium of Materia Medica” originated from the 16th century in the Ming dynasty and listed thousands of natural herb medicines described in detail. Maggot therapy can accelerate the removal of necrotic tissue and recovery of wounds, which shortens the treatment process of patients with diabetic foot (Bazalinski et al., 2022). Recent research studies have shown that the chemical composition of a maggot is made up of protein, fatty acid, chitin, etc (Taowen et al., 2022). The clinical application of the maggot is still widely concerned, although maggot standards continue to be improved. Pharmacological effects were also confirmed such as antimicrobial acerating, wound healing, blood glucose and lipid lowering, anti-inflammatory, immune regulation, and tissue reconstruction (Wang et al., 2020b, 2021; Lema et al., 2022; Shi et al., 2022). At present, the function of maggot extract (ME) on CAC still remains unknown, and whether ME pre-administration plays a chemo-preventive role in the initiation and development of CAC has not been reported.

Here, we focused on the preventive effects of ME by intragastric treatment prior to azoxymethane and dextran sulfate sodium (AOM/DSS)-induced CAC. In addition, the possible mechanism was investigated from the aspects of intestinal barrier repairing, inflammatory factor decreasing, and fecal microbial composition changing in the CAC model. These alterations were coupled and related to fecal non-targeted metabolic substance variation. Integrative analysis was used to clarify the relationship between gut microbiota and fecal metabolites in the divided groups.

## 2. Materials and methods

### 2.1. Animals and ethical considerations

**Figure 1:**
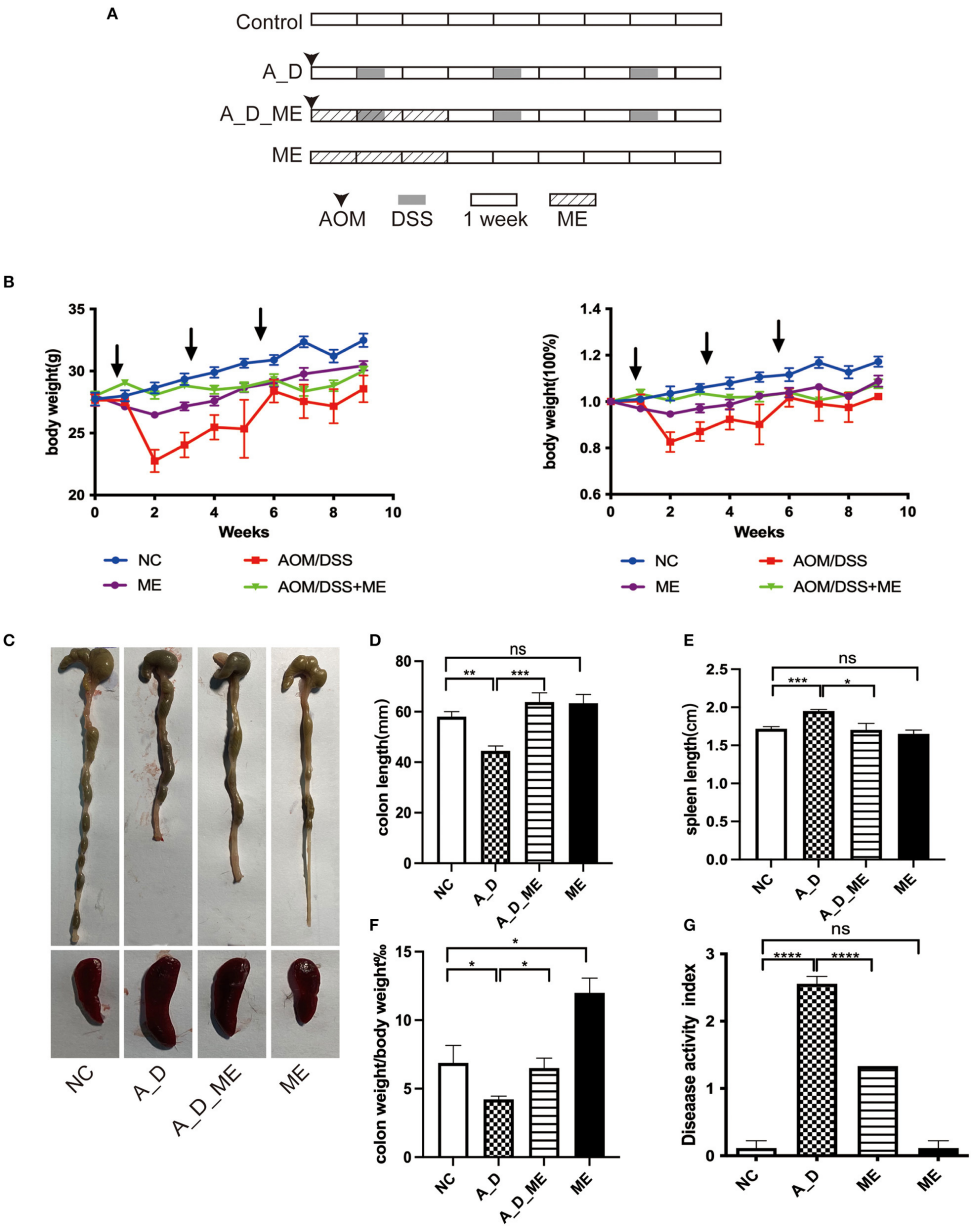
Effects of ME administration on the development of AOM/DSS-induced tumorigenesis in C57BL/6 mice (*n* = 6). **(A)** protocol, **(B)** body weight changes and the ratio of weight changes to the starting weight, **(C)** macroscopic morphologies of colon and spleen, **(D)** colon length, **(E)** spleen length, **(F)** the radio of colon weight to body weight, **(G)** DAI.

The experiment was performed on male C57BL/6 mice approximately 6 weeks old (Model Animal Research Center of Nanjing University), housing in a specific pathogen-free (SPF) environment (temperature 22±2°C; controlled humidity 50%;12/12 h day/night cycles). Ethical approval was listed by the animal ethics committee with a license. Animals were randomly assigned into four experimental groups (*n* = 6 in each group), as shown in Figure 1A: (a) normal control: sterile water daily, (b) the CAC model: azoxymethane (AOM) and dextran sulfate sodium salt (DSS), (c) ME pre-administration in model mice AOM/DSS plus ME administration consecutively for 21 days, and (d) only ME administration consecutively for 21 days.

Animals were cohoused for 1 week before the experiment. The control group drank sterile water every day. For the ME administrated group, the individuals were given (i.g.) 1 g/kg every day for 21 days from the first day of the experiment (day 1). The model mice were also injected intraperitoneally (i.p.) with 12.5 mg/kg of AOM (Sigma–Aldrich) per mouse on the first day (day 1). After a week, 2.5% DSS (MW36-50 kDa, MP biomedicals, United States) was given in drinking water for 5 days in the first cycle, followed by regular sterile water for the next 2 weeks. If mice lost 35% of their body weight, DSS can be replaced by drinking water, and then, DSS was allowed to continue for extra 2–5 days after mice were recovered from the loss. The cycle was repeated two times (5 days of 2%DSS), and all mice were then sacrificed 2 weeks later since the last cycle.

The colon was separated longitudinally, washed with phosphate buffer saline (PBS), and fixed as a Swiss roll in 4% paraformaldehyde after counting the number and size of polys in a blind trial. The specimens were embedded in paraffin, and serial sections were stained by HE (Hematoxylin–Eosin staining), IHC (immunohistochemistry), or IF (immunofluorescence). Blood, colon, liver, spleen, lung, and kidney tissues were collected for further experiments. During the experiment, the weight of mice was measured weekly, and stool was collected two times a week. The damage of disease was scored by the disease activity index (DAI), including weight loss, stool consistency, and bleeding. The scores were recorded after dividing the sum of subfractions by 3 and ranged from 0 to 4 individually.

### 2.2. ME preparation

The preparation and dose of ME were performed based on previous articles (Wang et al., 2019, 2021). In brief, blowflies were fed from larvae to maggots on wheat seeds, powdered milk, and yeast extracts. Large amounts of fresh maggot were flushed three times with water. After freeze-drying, maggots were ground into powder. Then, water solutions were obtained from PBS addition (twice the volume of the power), and the supernatant was collected by centrifugation at 15,000 r/min for 10 min after water-bath processing. Finally, the solutions were filtered through a 0.22μm membrane. The ME stock solution (500 mg/ml) was obtained.

### 2.3. Histological analysis (HE, IHC, and IF)

Tissues were prepared for sections (5μm), stepwise (200μm) through the paraffin block. The slides were dehydrated by gradient alcohol and stained with HE. The colon tissue of epithelial injury, inflammatory infiltration, and dysplastic hyperplasia was evaluated by pathologists separately.

As described above for IHC, the slides were blocked with 5% BSA for 1 h and incubated with primary antibody against ZO-1 (GB111402, Servicebio, China) and occluding (27260-1-ap, Proteintech, China) overnight at 4°C followed by incubation with secondary antibody for 1 h at room temperature. The sections were stained with DAB and then counterstained with hematoxylin.

To stain immunofluorescence, the experiment was carried out as mentioned above until the antigen is retrieved by citric acid buffer (PH6.0) through the microwave. Then, the slides were incubated with primary antibody overnight at 4°C after blocking. The secondary antibody conjugated with Alexa Flour 488 or 594 was used to incubate with the slides for immunofluorescence and DAPI for nuclei. The stained specimens were scanned by the laser scanning confocal microscopy (Leica DMIRE2, Germany).

### 2.4. Enzyme-linked immunosorbent assay (ELISA)

IL-1β and IL-6 in serum obtained on the last day were measured by mouse ELISA kit (Solarbio, China). The assays were executed according to the manufacturers' instructions. The absorbance of the specimen was detected at 450 nm by a microplate reader.

### 2.5. Western blot analysis

Colon tissue was cut and stored at −80°C in RIPA buffer (Beyotime, China) mixed with phosphatase inhibitor (Thermos Scientific, CA, United States) and protease inhibitor cocktail (Thermos Scientific, CA, United States). The lysates were centrifuged at 4°C (12,000 rpm, 20 min), and the supernatant was obtained. Protein quantification was performed by Enhanced BCA Protein Assay Kit (Beyotime, China). Proteins (30μg) per sample were used for Western blot analysis.

### 2.6. Quantitative Real-Time PCR assay

Tissue Total RNA Isolation Kit (Vayzme, China) was used to extract total RNA according to the protocol. Reverse transcription was performed to synthesize cDNA using PrimeScriptTM RT Master Mix (Takara, China), and then, cDNA was used. The primer sequences were shown in our previous studies (Wang et al., 2019). The relative expression of target mRNA was normalized by GAPDH and calculated a 2-ΔCt after obtaining a mean ΔC value. All results in triplicate were repeated three times.

### 2.7. 16s DNA sequencing

DNA was extracted from 200 mg stool of each sample. Specific primers with barcodes were used to amplify the conserved regions (V3-V4 region) (Guo et al., 2017) of ribosome RNA (rRNA). The primers were listed as follows: forward 5′-CCTACGGGNGGCWGCAG-3′ and reverse 5′- GGACTACHVGGGTATCTAAT-3′. The true PCR amplification products, with an average length of 466 base pairs, were recovered from the gel and quantified by the QuantiFluorTM fluorometer. The purified products were mixed in equal volumes and connected with sequencing adaptors to construct a sequencing library on the Illumina PE250 platform by Gene Denovo Biotechnology Co., Ltd (Guangzhou, China).

### 2.8. Bioinformatics processing

Sequencing reads were filtered to remove low-quality reads by FASTP (Chen et al., 2018). The rest of the reads was spliced paired-end with FLASH (version 1.2.11) and concatenated to create raw tags (Magoc and Salzberg, 2011). Then, raw tags were assembled, and clean tags were extracted (Bokulich et al., 2013). After strict quality checks, clean tags were clustered, and the chimeric tag (Edgar et al., 2011) was possibly removed by the UCHIM algorithm in USEARCH version 9.2.64 software (Supplementary Table S1). The Greengene database (version gg_13_5) was used as standard reference data (DeSantis et al., 2006). Finally, effective tags were obtained, and the abundance of operational taxonomic units (OTUs) was analyzed based on the effective tags by using USEARCH software (Edgar, 2013). Qiime (version 1.9.1) was used to estimate alpha and beta diversity indices (Caporaso et al., 2010) on a thin table of OTUs. The Shannon and Simpson metrics and ACE and Chao1 estimators were analyzed. The information of structural differences among samples was summarized from weighted-unifrac distances by an unweighted pair-group method with arithmetic means (UPGMA) tree. The linear discriminant analysis effect size (LEfSe) was employed to analyze differences between the groups shown by linear discriminant scores. Maps visualizing the principal coordinates analysis (PCoA) plots from the weighted and unweighted unifrac distances were drawn in R with the ggplot2, labdsv, and vegan packages (Lozupone and Knight, 2005; Hoegh and Roberts, 2020; Gao et al., 2021a; Liu et al., 2021). The characteristics of the microbiome were displayed at the taxonomic levels of phylum and family. A combination of PICRUSt2 (phylogenetic investigation of communities by reconstruction of unobserved states) and the Integrated Microbial Genomes database was used to construct phylogenetic trees, predicting bacterial genomics. Functions were, then, predicted based on the gene families and abundances using the Kyoto Encyclopedia of Genes and Genomes (KEGG) pathways. For the multivariate patterns represented numerically, outlier data were eliminated to prevent interference with the analysis. Items of information from 20 samples (*n* = 5 per group) were incorporated into the research.

### 2.9. Metabolomics profiling: ultra high-performance liquid chromatography-mass spectrometry (UHPLC-MS)

The stool samples were frozen at −80°C before a UHPLC-MS analysis. Each sample (50 mg) was added to a precooled solution of methanol/acetonitrile/water (2:2:1, v/v/v), which was mixed by ultrasound at a low temperature for 30 min. After standing for 10min at −20°C, the mixture was centrifuged at 14,000 g, at 4°C for 20 min, and the supernatant was dried under vacuum. During mass spectrometry, 100 μl of aqueous acetonitrile solution (acetonitrile: water =1:1, v/v) per sample was added to redissolve thoroughly and centrifuged at 14,000 g,at 4°C for 15 min. Samples of quality control were performed by blending 10 μl of every sample and then profiling with the whole samples meanwhile. QC got involved termly and studied at intervals of five samples to check the repeatability of the whole analysis.

The derivative was injected into a UHPLC (1290 Infinity LC, Agilent Technologies) coupled to a quadrupole time-of-flight (AB Sciex TripleTOF 6600) for analyzing untargeted metabolomics profiling of 20 fecal samples. A 2.1 mm × 100 mm ACQUIY UPLC BEH 1.7 μm column (waters, Ireland) was employed for RPLC separation.

### 2.10. Metabolomics data mining

MS raw (.raw) documents were transformed into the mzML format by proteowizard and were analyzed in R with the XCMS package, consisting of retention time alignment, peak identification, and peaks matching. After the preprocessing of the data matrix, it was formed including mass-to-charge ratio, retention time, and peak area. Precursor molecules in positive and negative ion modes were accessed, and the molecules were normalized to obtain quantitative results. Identified metabolites were projected to KEGG pathways. The detailed descriptions of data mining and statistical analysis are presented in Supplementary material.

### 2.11. Statistical analysis

The Kruskal–Wallis test was accomplished by LEFse to value the differences among the microbiota compositions of the four compartments. Moreover, the selected differences were compared between any two groups by the Wilcoxon rank sum test. The final differences were ranked using the results of a linear discriminant analysis (LDA). The VIP value of multivariate statistical analysis of OPLS-DA was combined with the *P*-value of univariate statistical analysis in a T-test, screening the differential metabolites between different groups. The threshold of differences was as follows: VIP≥ 1 in the OPLS-DA as well as p < 0.05 in the *t*-test. The correlation between gut microbiota communities and fecal metabolites was analyzed by Pearson's correlation coefficients. The *p*-value was calculated based on Fisher's Z-transformation. Differences were statistically significant at *p* < 0.05.

## 3. Results

### 3.1. Effects of ME administration on the development of AOM/DSS-induced tumorigenesis in C57BL/6 mice

During a 15-week period in an AOM/DSS-induced tumorigenesis, weight loss was observed compared with the NC group, particularly DSS water drinking in the 2nd, 5th, and 8th weeks. Upon changing from DSS to sterile water, the body weight was recovered. There were significant differences in the ME-treated group throughout the period of 21 days, but no differences were observed on the day of sacrifice (Figure 1B). ME treated for 21 days in mice proved no toxicity in the aspects of gross abnormality and serological indicators (Supplementary Figure S1). Except for the NC and ME groups, the mice in the rest of the two groups caused bloody stool and ulcers (not shown). Morphology was visibly altered in the terms of the colon and spleen (Figures 1C–E). As shown in Figures 1D, F, AOM/DSS caused length shortening and weight reduction in the colon tissue. However, the length and weight of the large bowel in ME-treated CAC mice were improved compared with those in the AOM/DSS group (*p* < 0.05). Obvious splenomegaly led by AOM/DSS was also visible while an effective reversal on the enlargement of the spleen was shown compared with the supplement of ME in the models. Compared with the control group, the DAI was scored on the last day, increasing in AOM/DSS-exposed mice, and pre-administration of ME showed improvement clearly (Figure 1G).

### 3.2. Decreasing severity of inflammation and carcinogenesis in AOM/DSS-treated mice after ME administration

**Figure 2:**
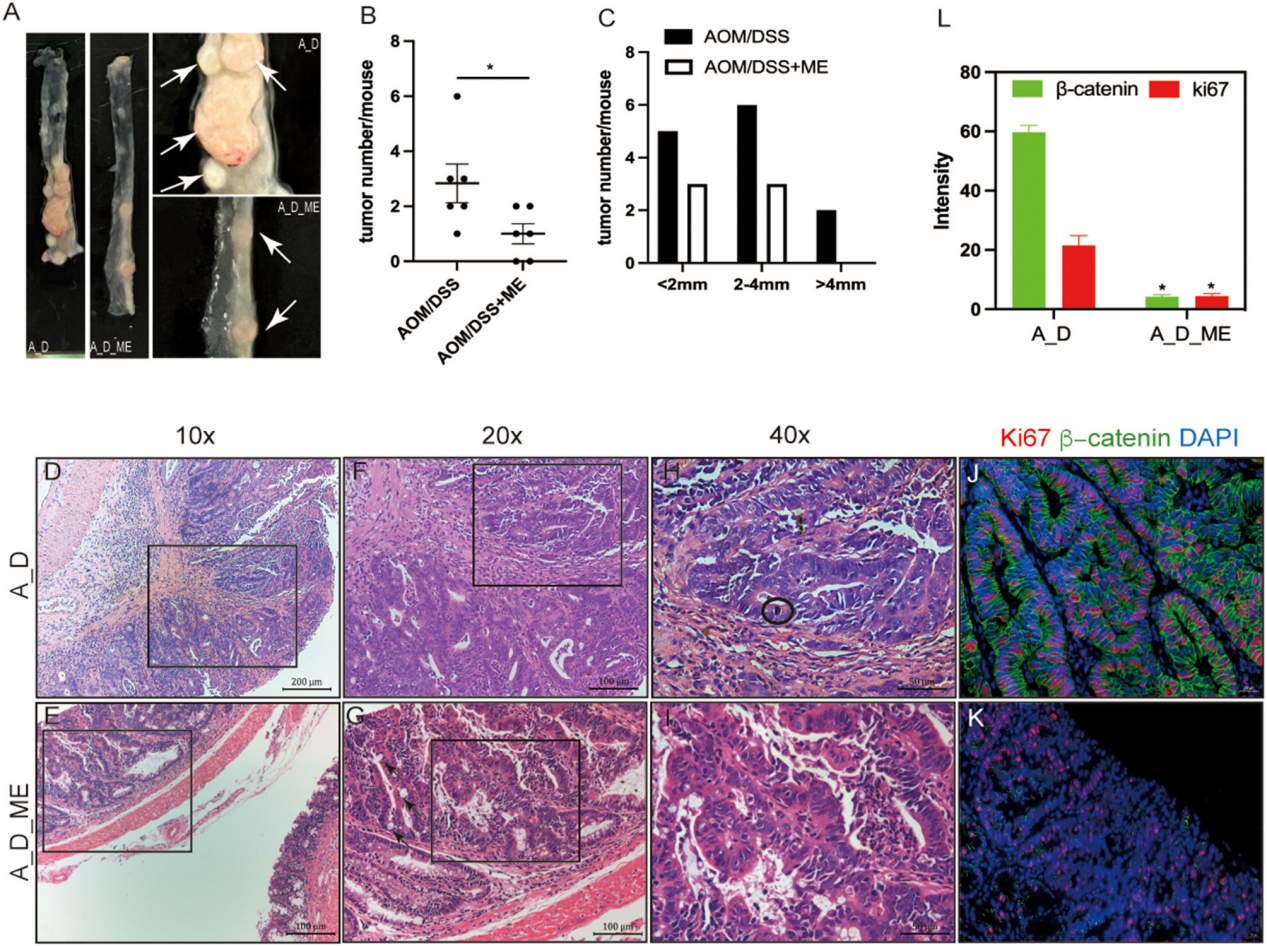
Decreasing severity of inflammation and carcinogenesis in AOM/DSS-treated mice after ME administration. **(A)** tumors formed in the large intestinal tracts. The white arrow represents tumors. **(B, C)** numbers and distributions of tumors in the AOM/DSS mice (*n* = 6 per group). **(D–I)** representative micrographs of H&E staining, **(D, E)** representative adenomas, infiltrating inflammatory cells in the surface of the rectum, scale bar=200μm, 10×magnification, **(F, G)** pathology damaged in the colonic mucosa, scale bar = 100μm, 20×magnification, the black arrow represents intestinal metaplasia, **(H, I)** degree and type of cell differentiation, evaluation of mitotic figure can be hard(encircled). scale bar=50μm, 40×magnification. **(J–L)** immunofluorescent staining of ki67 (red) and β-catenin(green) in the adenocarcinoma of AOM/DSS model treated with ME or not, scale bar=20μm, fluorescence intensity is analyzed by image J. Wilcoxon rank sum test was used, *p < 0.05.

To investigate the function of ME in CAC, the model we used was administrated by a dose of AOM and three cycles of DSS (Figure 1A). The repeated DSS-induced IBD, as a result of chronic inflammation, slightly increases the incidence of AOM-caused tumors. We noticed a dramatic reduction of approximately 67% in polypoid colonic tumor incidence in the ME pre-administration group (Figure 2B). These tumors were macroscopically located in the middle and distal colon (Figure 2A), where inflammation induced by DSS occurs severely, indicating that the severity of colitis was identified with the incidence of a tumor. We divided the tumors into three types: 0–2, 2–4, and >4 mm; a larger diameter means the cancer is more serious. Significant differences in size between tumors in model mice and ME-treated models could be detected, indicating that ME pre-administration alleviates inflammation-associated carcinogenesis in the model colon (Figure 2C). The tumors were largely adenomas with low-grade or high-grade differentiation in intraepithelial neoplasia and different degrees of inflammatory cell invasion (Figures 2D–I, Supplementary Figures S2A, B). A decreasing expression of β-catenin and ki67, very representative markers in colorectal carcinogenesis, was observed in a colonic crypt in the ME-treated group (Figures 2J–L).

### 3.3. Effects of ME administration on the regulation of tight junction proteins and inflammatory responses in AOM/DSS-treated mice

**Figure 3:**
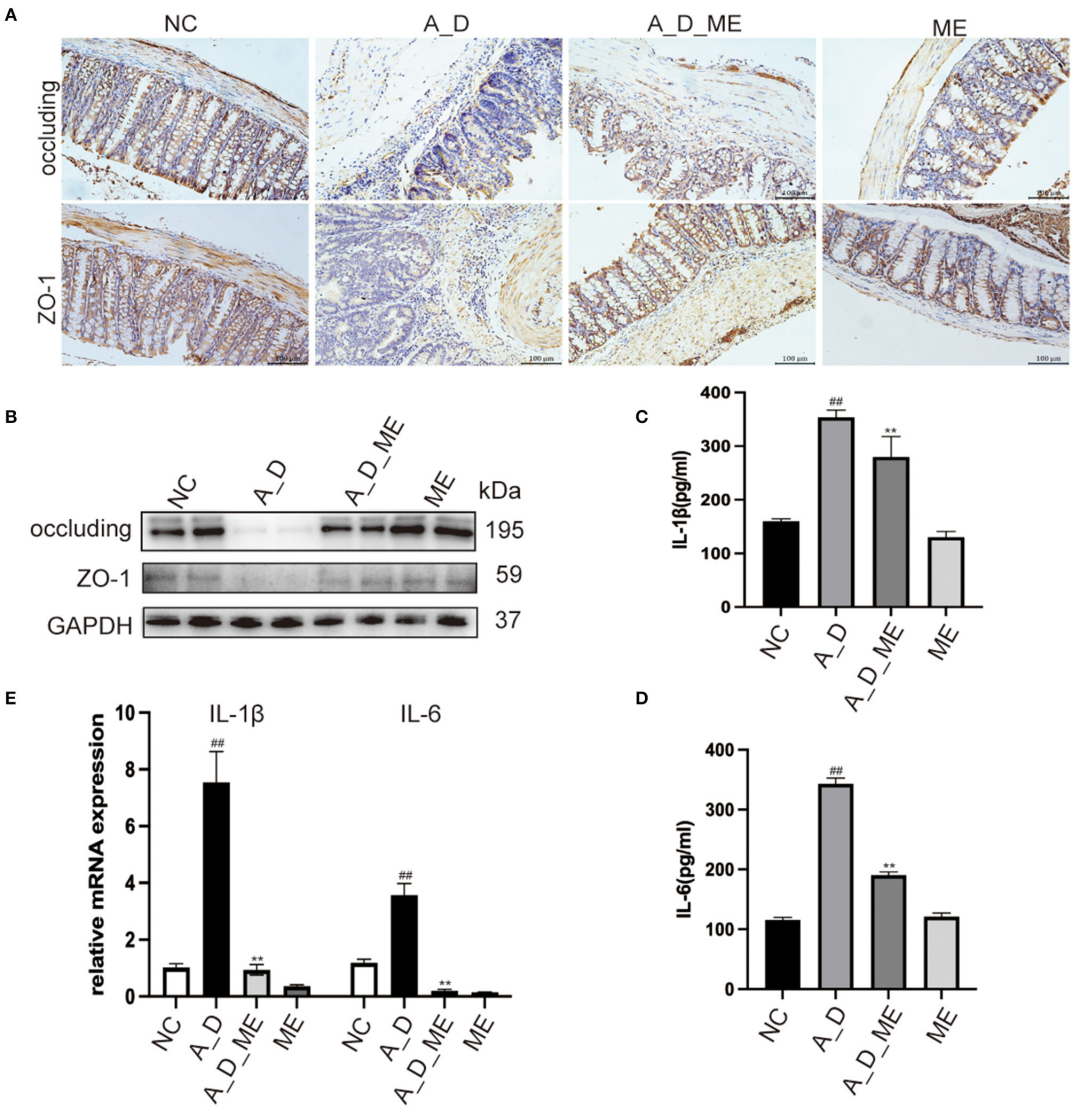
Effects of ME on the regulation of tight junction proteins and inflammatory responses in AOM/DSS-treated mice. **(A)** representative IHC staining of occluding and ZO-1 in treated mice as expressed. Scale bar = 100 m. **(B)** the expression of occluding and ZO-1 in colon tissues was detected by Western blotting. Graphs were quantified using GAPDH as the internal reference. **(C, D)** the serum levels of IL-1β as well as IL-6 in four groups. **(E)** the relative mRNA levels of IL-1β as well as IL-6 in the colon tissues. Data represent the mean ± SD, ^##^*p* < 0.01 compared to normal control, **p < 0.01 compared with the model group. *N* = 6/group.

Pre-administration of ME was found to reverse the downregulation of zonula occluden-1 (ZO-1) and occluding, which were significantly reduced in the AOM/DSS group (Figures 3A, B). As a result, we suggested that the effects of ME on the CAC model mice were in connection with the regulation of ZO-1 and occluding, functioning in the aspects of the intestinal mucosal barrier homeostasis. The reports showed that CAC had a sign with the production of a variety of inflammatory factors such as IL-1β and IL-6. Our ELISA results demonstrated that the serum levels of IL-1β and IL-6 expanding in mice treated with AOM/DSS were decreased by ME (Figures 3C, D). Meanwhile, it was also shown that after ME administration, the impression of AOM/DSS on CAC was partially offset in the mRNA expression of IL-1β and IL-6 in colonic tissues (Figure 3E).

**Figure 4:**
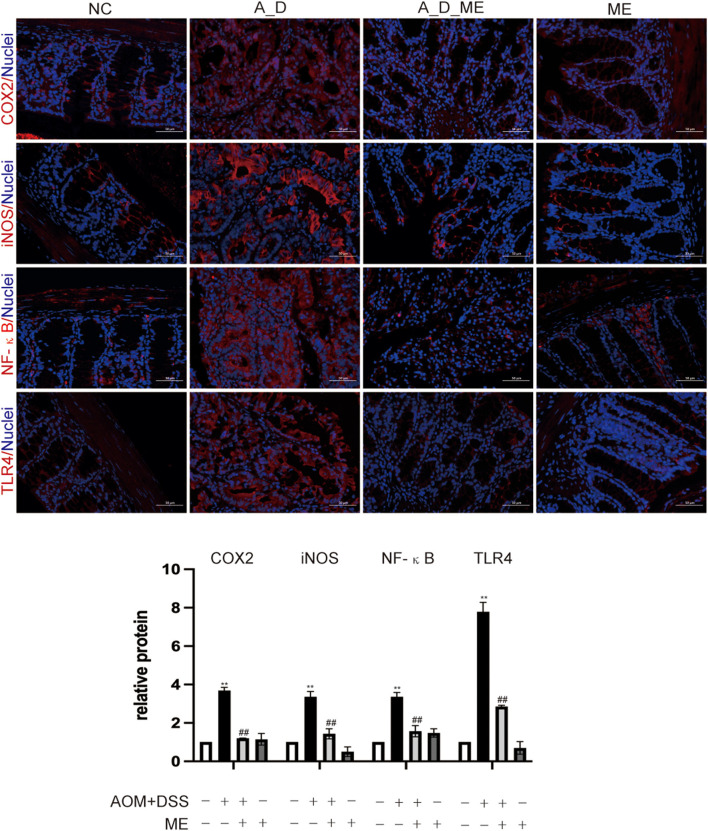
Aberrant regulation of TLR4 and NF-κB signaling pathway in the large intestinal tissues. COX2, iNOS, NF-κB, and TLR4 were assessed by immunofluorescence staining of the mice colonic slides in four groups (400× magnification). Histogram showing the percent of positive staining in the tissue. Data represent the mean ± SD, ^##^*p* < 0.01 compared with normal control, **p < 0.01 compared with the model group. *N* = 6/group.

Colitis-associated carcinoma has proven to be a complicated process. In AOM/DSS-induced mice, Toll-like receptor 4 (TLR4) mediated intracellular nuclear factor-κB (NF-κB)-containing signaling cascades, encouraging the progression of cancer. After NF-κB was activated, it leads to the release of pro-inflammatory mediators including interleukin-1β (IL-1β), interleukin-6 (IL-6), inducible nitric oxide synthase (iNOS), and cyclooxygenase-2 (COX-2). Our results of immunofluorescence staining exhibited that the positive cells of TLR4, NF-κB, iNOS, and COX2 showed the highest expression in the colon tissue of the AOM/DSS group. However, we observed that the levels of TLR4, NF-κB, iNOS, and COX2 in the ME pre-administrated group showed lower expression than that in the AOM/DSS group. Data revealed that the increasing expression of TLR4, NF-κB, iNOS, and COX2 in the AOM/DSS group was inhibited by ME treatment (Figure 4).

### 3.4. Mice treated by ME and fecal microbiome

**Figure 5:**
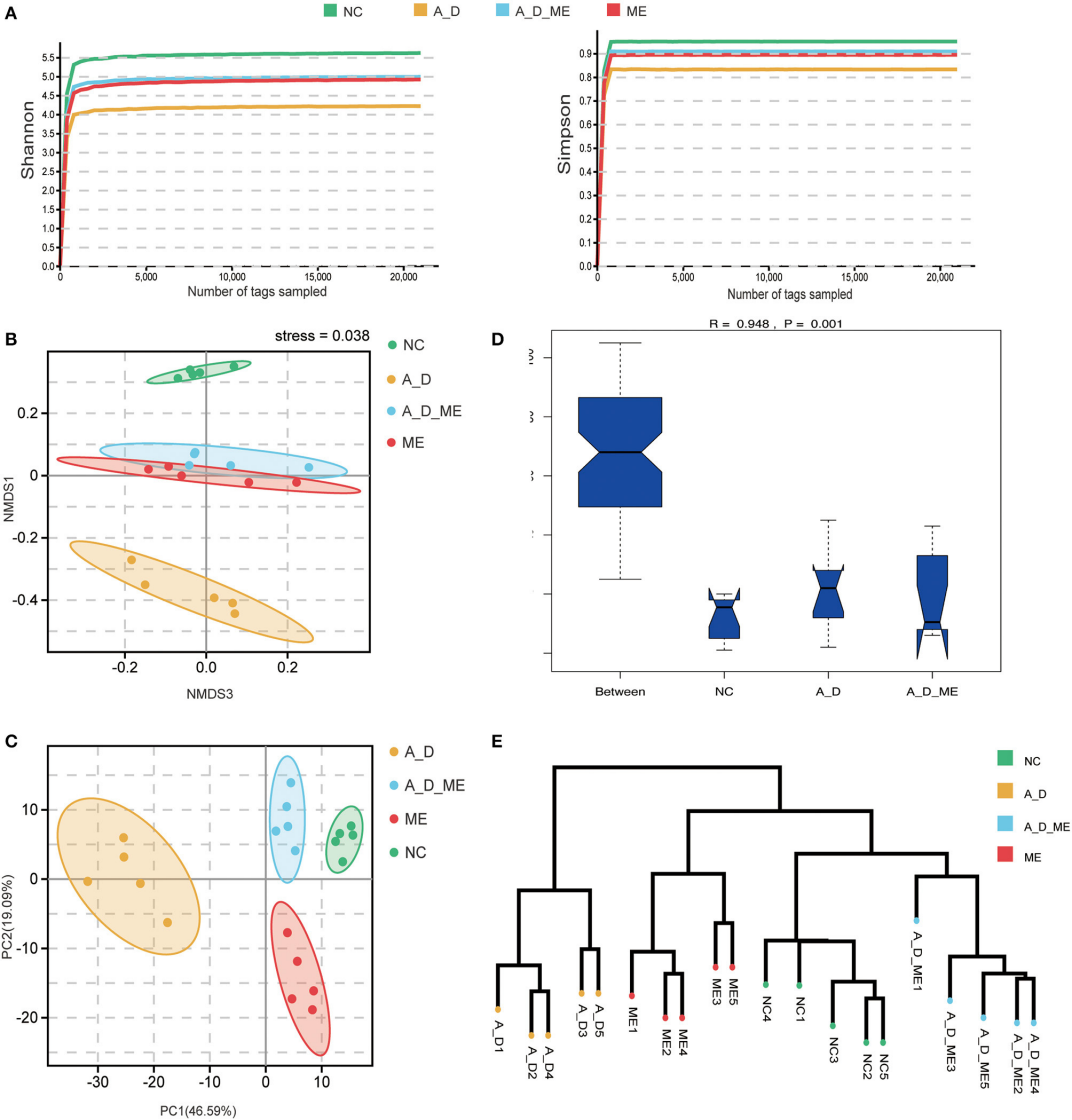
Effects of ME intragastric administration on microbial diversity in intestinal fetal, decided by 16s rRNA sequencing. **(A)** rarefaction curves of Shannon and Simpson index, **(B, C)** plots of nonmetric multidimensional scaling (NMDS) analysis, and Principal Component Analysis (PCA) based on weighted unifrac distance. The more similar the sample had, the closer the distance reflected in the plot. **(D)** boxplot of analysis of similarity (ANOSIM) based on weighted_unifrac among the three groups, NC vs. A_D R = 0.996 *p* < 0.05*, A_D vs. A_D_ME R=0.776, **p < 0.01, **(E)** UPGMA clustering method of all samples was classified on the basis of the unifrac-distance matrix, R=0.948, **p < 0.01. The more similar samples had shorter common branches.

To investigate the association between ME's impact and intestinal microbiome, we focus on the composition of the fecal bacterium by 16s rRNA sequencing. After extracting clean reads and producing effective tags, high-quality sequencing and quality control were used for subsequent taxonomy analysis (Supplementary Table S1). The multi-sample rarefaction curves of Shannon and Simpson indices tended to be smooth when the sample tags added up to approximately 2,000, indicating an extensive sequencing depth and the most captured diversity for fecal microbiome analysis (Figure 5A). Moreover, Simpson's results were similar to Shannon's. Microbial community alpha diversity metrics (Shannon, Simpson, Chao, Goods' coverage, Pielou, and pd shown in Supplementary Table S2) and beta diversity indices (NMDS and PCA, shown in Figures 5B, C) were significantly different between the groups with and without ME (NC vs. ME, A_D vs. A_D_ME). The NMDS and PCA plots of weighted unifrac_distances were clearly separated observing by ME status. The obvious shift of the ME group was narrowed compared to the control (A_D vs. A_D_ME compared to NC vs. A_D). Moreover, ANOSIM analysis showed that the effects among the three groups were also significantly different (Figure 5D). The unweighted pair-group method with arithmetic means (UPGMA) clustering was likewise employed to access the beta diversity of gut microbiome among groups (Figure 5E). The UPGMA method divided the individuals into the A_D group and the other groups, suggesting that the microbial profile was definitely diverse between the model mice and ME-treated model mice. There was a certain degree of similarity in the NC group and A_D_ME group, which reveals that ME administration reversed the microbial profile of the AOM/DSS model mice. Together, ME had obvious effects on the alpha and beta diversities of the gut microbiome.

**Figure 6:**
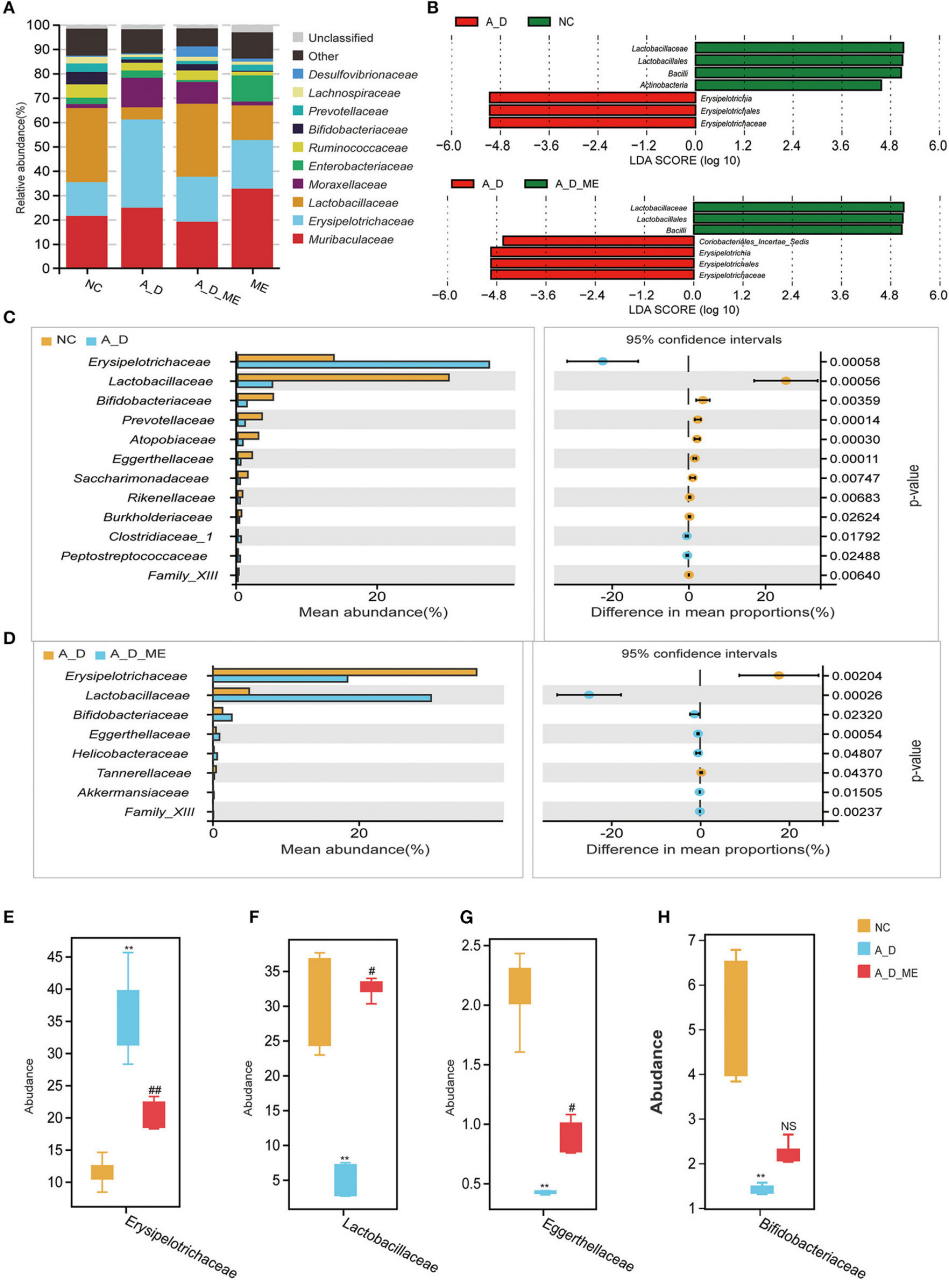
Effects of ME intragastric administration on the proportion of intestinal microbiota at the family level. **(A)** Each column in the histogram shows the relative abundance for one group and detailing the top 10 species among the mean abundance of each sample. The remaining unknown species and unclassified sequencing data were separately marked as “Other” and “Unclassified”. **(B)** differences in biomarkers by means of taxonomic line discriminant analysis (LDA) effect size (LEfSe) method among the NC, A_D, A_D_ME groups. LDA scores indicated by the bar graph represent the effect of the different species at the family level. **(C, D)** differences of abundance at the family level in the NC group compared with the A_D group **(C)** and the A_D group compared with the A_D_ME group **(D)**, Welch's t-test is used to identify the difference between the two groups. Differentially abundant family according to ME administration. **(E–H)** Box plots are shown by mean data (SD) of the abundance ratio of four families (*Erysipelotrichaceae, Lactobacillaceae, Eggerthellaceae, and Bifidobacteriaceae* among five individuals in every three groups. Tukey's HSD test is used to analyze the differences. *N* = 5/group, * *p* < 0.05, *vs*. NC, **p < 0.01, *vs*. NC; # *p* < 0.05, *vs*. A_D, ## *p* < 0.01, *vs*. A_D, ns means no significance.

According to operational taxonomic units (OTUs) identified from sequenced samples, the most relative abundances in the level of phylum were *Firmicutes, Bacteroidetes, and Proteobacteria* (Supplementary Figure S3). At the family level, the microbial profile of the NC group, A_D group, A_D_ME group, and ME group belonged to the most 10 common families as follows: *Muribaculaceae, Erysipelotrichaceae, Lactobacillaceae, Moraxellaceae, Enterobacteriaceae, Ruminococcaceae, Bifidobacteriaceae*, and other three families (Figure 6A). LEfSe analysis showed that CAC mice treated by ME had a correlation with rich abundances of *Lactobacillaceae and Bacilli* and poor abundances of *Erysipelotrichaceae* and Coriobacteriales_Incertae_Sedis (Figure 6B). Welch's t-test was used to analyze the top biomarkers in the taxa that could identify the NC group, A_D group, and A_D_ME group (Figures 6C, D). A total of three probiotics (*Lactobacillaceae, Bifidobacteriaceae*, and *Eggerthellaceae*) decreased in A_D but increased in the NC and A_D_ME groups, while another pathogen (*Erysipelotrichaceae*) increased in A_D but decreased in the NC and A_D_ME groups (Figures 6E–H). Therefore, ME-associated families including *Lactobacillaceae, Eggerthellaceae, Erysipelotrichaceae*, and *Bifidobacteriaceae* were incorporated into the following analysis. The *Lactobacillaceae* and *Erysipelotrichaceae* families were classified into the same and most phylum Firmicutes, while *Eggerthellaceae* and *Bifidobacteriaceae* were classified into the phylum Actinobacteria.

### 3.5. Mice treated by ME and fecal metabolomic profile

**Figure 7:**
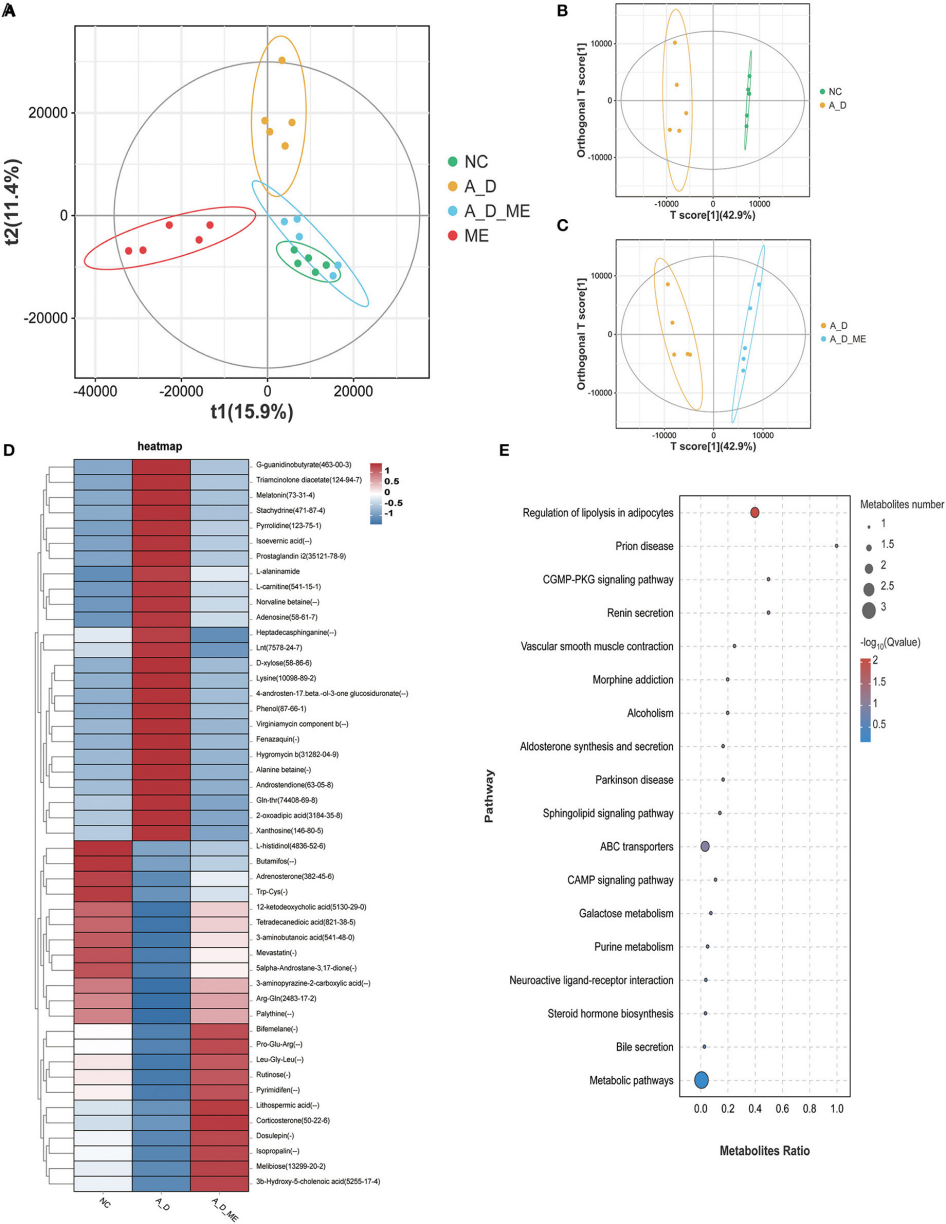
Alteration of ME administration on metabolomics in the fecal. score scatter plots for (PLS-DA) the model showed that almost all of the data is in 95% hotelling's T-squared ellipse. The X and Y axes are the scores of the first and second principal components **(A)**. Combining orthogonal signal correction (OSC) and PLS-DA, the X matrix information can be decomposed into two types of information related to Y and irrelevant. By removing the irrelevant differences, the relevant information is concentrated in the first predictive component (the predicted score of the X-axis). The Y axis represents the score of the main orthogonal component. An orthogonal projection to latent structures-discriminant analysis (OPLS-DA) model was employed to analyze and screen differential metabolites. NC vs. A_D **(B)**, A_D vs. A_D_ME **(C)**. **(D)** heatmap of 48 differential metabolites enriched in the three groups, **(E)** bubble map of KEGG enrichment pathway. The top 20 pathways with the lowest Q value are used to draw the map. The X axis is the pathway and the Y axis is the ratio of the metabolites (the number of differential metabolites in the pathway divided by all the numbers in the pathway). The size stands for the number and the color stands for the Q value. *N* = 5 mice per group.

Metabolite differentiation was presented among the treated groups by the Partial least squares-discriminant analysis (PLS-DA), and all four groups were almost separated (Figure 7A). An orthogonal projection to latent structures-discriminant analysis (OPLS-DA) also revealed an obvious distinction between NC and A_D mice and between the A_D group and A_D_ME group (Figures 7B, C). As shown in Figure 7D, a total of 48 fecal metabolites in the relevant three groups were listed. The results demonstrated that the relative abundances of presented fecal metabolites in CAC mice were quite different from those in the control group, implying that AOM/DSS treatment has a profound and lasting influence on fecal metabolic profiles. ME administration in CAC mice also showed significant differences in the abundance of fecal metabolites. The changes in the listed 25 metabolites caused by AOM/DSS treatment were attenuated by ME administration, including G-quanidinobutyrate, Triameinalana dissatsta, Stachvdrine, Isoevernic acid, Prostaglandin i2, and I-alaninamide. In addition to that, the abundances of 12-ketodeoxycholic acid, Tetradecanediodic acid, 3-aminobutanoic acid, 5alpha-Androstane-3,17-dione, 3-aminopyrazine-2-carboxylic acid, Arg-Gln, and Palythine were decreased in the model mice, whereas the alterations were diminished after ME administration.

To evaluate the importance of 48 chemical compounds, metabolite pathways were analyzed, involved in the regulation of lipolysis in adipocytes, prion disease, the CGMP-PKG signaling pathway, renin secretion, vascular smooth muscle contraction, morphine addiction, alcoholism, and aldosterone synthesis and secretion (Figure 7E).

### 3.6. Correlations between host fecal microbiota and metabolites

**Figure 8:**
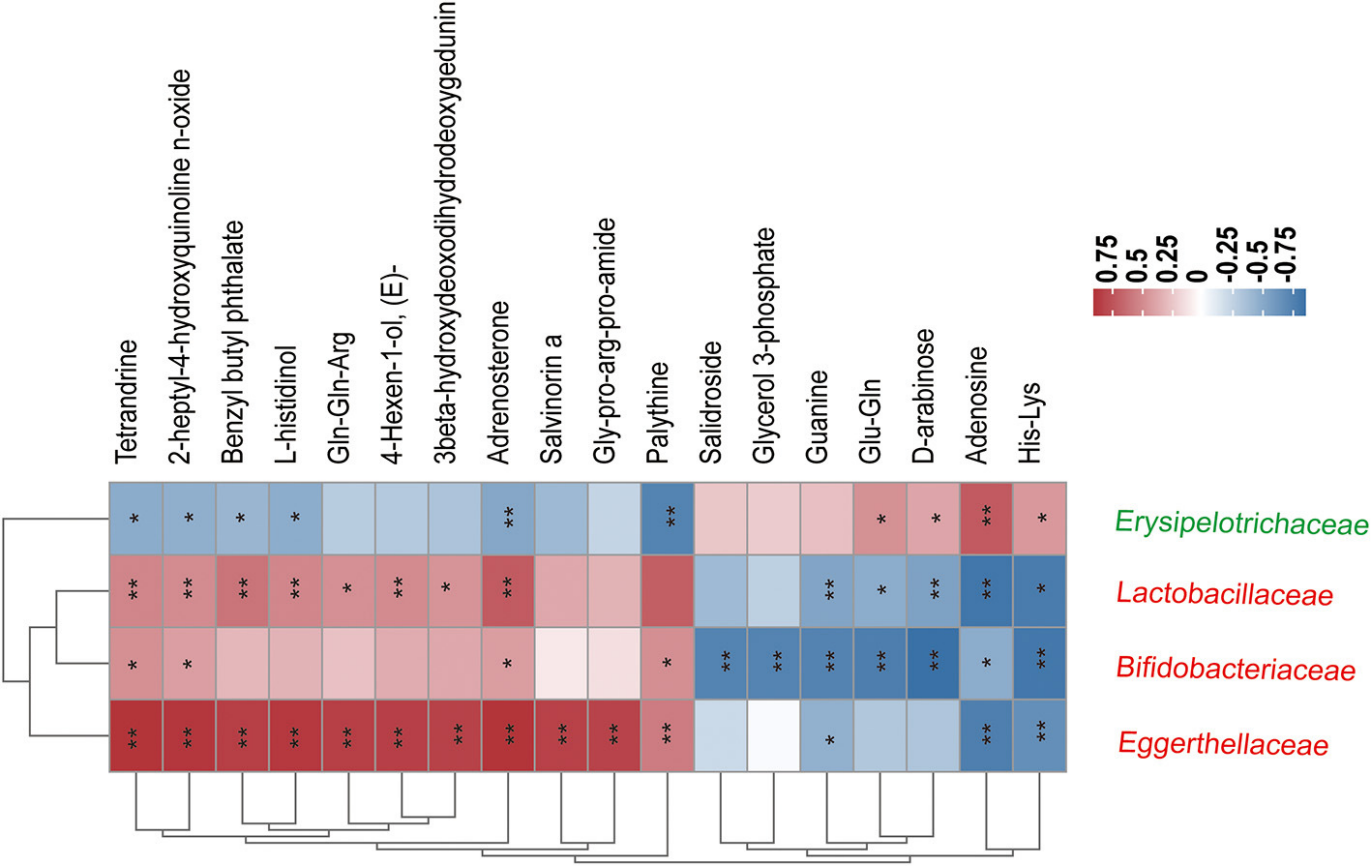
Correlation coefficients between the relative abundance of four ME-associated bacterial families and the top 20 fecal metabolites in the treated groups. The color depth of the grids depicted the strength of the correlation (red square= positive correlation, blue square = negative correlation. *p < 0.05, **p < 0.01). Increased and decreased relative abundance in AOM/DSS mice treated by ME were denoted by red and green tags, respectively.

As shown in Figure 8, correlations between 4 ME-associated bacterial families and the top 20 of all the altered metabolites in the fecal were analyzed. For example, fecal Tetrandrine, Adrenosterone, and 2-heptyl-4-hydroxyquinoline n-oxide had positive relations with three ME-increased bacterial families, notably *Lactobacillaceae, Bifidobacteriaceae*, and *Eggerthellaceae*, but negative relations with ME-decreased bacterial family, *Erysipelotrichaceae*. In addition, *Erysipelotrichaceae* was not related to Gln-Gln-Arg, 4-Hexen-1-ol, 3beta-hydroxydeoxodihydrodeoxygedunin, Salvinonin a, Gly-pro-arg-pro-amide, Glycerol 3-phosphate, Salidroside, or Guanine, except for a positive relation with Gln-Gln, D-arabinose, Adenosine, and His-Lys. The data showed that host gut microbiota composition was identified to work on fecal metabolites.

## 4. Discussion

CRC fatality keeps rising worldwide, sometimes CRC is caused by genetic or familial basis (Siegel et al., 2020), but seldom the results from exclusively intestinal inflammation which was a double-edged sword in tumorigenesis (Schmitt and Greten, 2021). Though approximately 20% of IBD will be with CAC, more than 50% of them die of CAC, and it is one of the most worrisome complications of IBD (Nadeem et al., 2020). Data obviously showed that anti-inflammatory treatment with drugs was practical in preventing or alleviating colon cancer stages (Malik et al., 2018). As a result, it is urgent to develop precision medicine that delay or even stop the conversion from inflammation to cancer, as well as improve living quality with low toxicities. According to this strategy, ME was chosen as a new bioactive insect, owing to its multiple biological activities, especially its anti-inflammatory effects (Wang et al., 2021; Lema et al., 2022). Bioassay-guided fractionation was used to isolate antibacterial substances from the secretions of the living maggot body (Gao et al., 2015). The peptide compounds of maggot had been confirmed to influence the treatment of diabetic foot (Taowen et al., 2022). Medical maggots were also undertaken by therapeutic nurses at the ocular surface, with the attribution to the validation of peptide compounds (Bazalinski et al., 2022; Lema et al., 2022). Polysaccharide substances were extracted from the maggot body and induced the composition of GMB in high-fat diet mice (Wang et al., 2020b; Shi et al., 2022). Additionally, ME ameliorated intestinal fibrosis in DSS-induced chronic colitis (Wang et al., 2021). Our previous study reported the anti-cancer effects of ME in human ovarian cancer cells (Wang et al., 2022a). Moreover, its powder was investigated for therapeutic function by interrupting bacterial biofilm (Becerikli et al., 2022). The toxicity of altered intestinal and other major organs after ME administration was unlikely to appear because ME had no significant influences on clinic indicators and morphological features (Supplementary Figure S1).

In the present research, one of the key findings was that ME had superior positions in ameliorating AOM-induced and DSS-induced splenomegaly, colon length reduction, intestinal barrier damage, as well as inflammation of colon cells in mice. The results also inferred for the first time that ME revealed ideal prevention of intestinal dysbiosis in CAC mice, accompanied by and correlated with alterations in the composition of metabolites. Although specific ingredients of ME were not evaluated in this research, it still deserves further study.

The observed mucosal barrier changes were similar to previous reports on AOM/DSS-induced enteropathy (Li et al., 2019; Oh et al., 2020; Luan et al., 2022), mainly featured by losing the expression of ZO-1 and occluding and blooming release of inflammatory factors, IL-1β and -IL6 (Figure 3). In previous studies, DSS activated the TLR4-mediated signal pathways, afterward NF-κB phosphorylation cascade, to regulate the feedback of inflammatory factors, which was correlated with the tight junction protein (Sinha et al., 2020; Jin et al., 2021). This explained the increased levels of ZO-1 and occluding in a way, resulting from the participation of factors in promoting intestinal epithelial permeability. Since the activation of NF-κB leads to increasing transcription of abundant genes, that functioning in the aspects of immune responses, proinflammatory effects and cell apoptosis. (Jayandharan et al., 2011; Bessa-Goncalves et al., 2020). Indeed, one can predict that in some types of cells, the activated NF-κB may enhance tumor development but inhibit tumor incidences in other types (Gao et al., 2021b; Mirzaei et al., 2021). Interestingly, our results confirmed that the enterocyte proliferation (ki67 and β-catenin) was reduced after the administration of ME, associated with less tumor burden (Figure 2). ME reversed the expressions of intestinal mucosal barrier markers occluding and ZO-1 in the CAC model by repairing the TLR4 cascade and decreasing inflammatory genes, therefore slowing tumor progression (Figure 4).

It was worth noting that the enterocytes had diametrically opposed influences on LPS from gram-negative bacteria for a long time. Since LPS can stimulate TLR4, which leads to initial immune responses in enterocytes, the role of TLR4 mediating the development of colitis-associated tumorigenesis has been established in the aspects of enhancing direct recruitment of NF-κB and the large increase in cytokines (Park et al., 2009; Olona et al., 2021). One way to activate the interaction between TLR4 and NF-κB and thereby drive LPS-dependent proinflammatory progression may be important for the severity of intestinal inflammatory response. The reports have provided a few examples that TLRs, interacting with endogenous ligands from the host, especially TLR4, are engaged in the process of infectious and non-infectious diseases (Tan et al., 2015). The present study revealed that ME had a repressive response to TLR4 using a chemical carcinogenesis model, and its inhibition suggested that TLR4-mediated NF-κB exerted an effect on tumor burden reduction. Consequently, downregulated expression of NF-κB contributed to the inhibition of COX-2 and iNOS (Figure 4). Wang et al. reported that ME repressed the Nrf2/NF-κB signaling pathway, including the production of downstream kinases in DSS-induced colitis (Wang et al., 2019, 2021). Furthermore, iNOS is an important enzyme and produces some compounds, involved in oxidative stress and inflammatory response (Cinelli et al., 2020). As far as we know,COX-2 catalyzes arachidonic acid into prostaglandin, acting as a mediator that cause pain or inflammation. The inhibitor of COX-2, such as 5-ASA and aspirin both successfully supported in chemoprevention, has been proven to be a key point to alleviate colonic inflammation even with CRC occurrence (Burn et al., 2011; Kaur et al., 2020). In this study, despite increasing productions of iNOS and COX-2 induced by AOM/DSS, ME pre-administration suppressed their proteins in the colon tissue of model mice, suggesting that ME could reduce chronic inflammation-associated tumor initiation by the inhibition of TLR4, NF-κB, iNOS, and COX-2. The relationships of TLR4- mediated NF-κB signal pathway in intestinal cells have been supported in many research studies; thus, various mechanisms have been established (Jin et al., 2021). One question still remains unclear that which types of cells act for ME-reversed inflammatory hallmarks in CAC.

Gastrointestinal cancer has proven to have connections with intestinal flora (Janney et al., 2020; DeDecker et al., 2021). Moreover, chronic inflammatory response and intestinal barrier damage might be related to dysbiosis in the gut flora as described above. Consistently, the enteropathy will tend to be recovered to normal condition via administration with probiotic bacteria directly (Suez et al., 2019; Samara et al., 2022). This study focused on the changing microbiome caused by ME pre-administration for 21 consecutive days in the initial phase of the model. At the end of the period, 16S rRNA analysis showed the improving relative abundance of the intestinal bacterium in the model treated with ME, and then, we identified four microbial families, namely *Erysipelotrichaceae, Lactobacillaceae, Bifidobacteriaceae*, and *Eggerthellaceae*, the last three probiotics of which were dramatically enriched (Figures 5, 6). The results also showed the inhibition of pro-inflammatory *Erysipelotrichaceae* and the acceleration of anti-inflammatory probiotics after ME administration. The family *Lactobacillaceae* has been reported to regulate the immune system, including the management of initial immune response, improvement of cellular and humoral immunity, and inhibition of pathogenic microorganisms (Lin et al., 2020). It has been reported that probiotics weaken the capability of proliferation in colon cells and prevented tumor migration or angiogenesis. Previous studies reported that pro-inflammatory cytokines, such as IL-1β, IL-6, IL-17, and IL-22, in the blood reduced significantly and were observed in a CRC patient trial with consumption of 6-month probiotics (IL-1 is required for tumor invasiveness and angiogenesis) (Samara et al., 2022).

The score plots of both PLS-DA and OPLS-DA revealed the changing metabolic profiles in fecal samples. Our study confirmed the disordered host microbiota and compositional-metabolomic fluctuations (48 chemical compounds included) after ME treatment, as a result of the prevention of CAC occurrence (Figures 7, 8). Metabolite pathways were also analyzed by topology programs, greatly differing between the A_D and A_D_ME groups. We hypothesized that pre-administration of ME could change the levels of metabolomics, contributing to the homeostasis of the gut microbiota. ME administration significantly increased products of fat metabolism, including 12-ketodeoxycholic acid, tetradecanedioic acid, 3-aminobutanoic acid, and 3-aminopyrazine-2-carboxylic acid in the fecal of AOM/DSS mice. Fecal secondary bile acids, 12-ketodeoxycholic acid included, were significantly higher during the ME supplementation periods. Data showed that secondary bile acids functioned in the regulation of cholesterol and lipid and the production of active oxygen and nitrogen (Reinicke et al., 2018; di Gregorio et al., 2021), as well as reducing the levels of cytokines engaged in inflammation (Sinha et al., 2020; Feng et al., 2022). Thus, we speculate that ME exacerbates bile acid metabolism and has an impact on gut homeostasis. Despite the role of Prostaglandin in CAC being controversial (Hirano et al., 2020), our analysis demonstrated that Prostaglandin I2 was significantly amplified in the model mice. In accordance with studies reported (Iwanaga et al., 2014; Wang et al., 2022b), Prostaglandin I2, as the product of Prostaglandin I synthase, was a chemo-preventive or antimitogenic agent in tumor angiogenesis or growth (Cathcart et al., 2010; Minami et al., 2015). Meanwhile, major urinary metabolic products of prostaglandin had been verified for its clinical benefits monitoring as a non-invasive biomarker in ulcerative colitis (Gao et al., 2021c). Notably, some metabolites (e.g., phenol in fecal samples) are most possibly toxic to bodies (Van Hecke et al., 2021). A significant loss of fecal phenol shown in the ME-treated and NC groups compared with model mice may be related to some bacterium shift in intestinal microbial composition. Adenosine, acting in many pathophysiological processes, potentially mediates the proliferation through mutual effects with receptors. Interestingly, we found the reversed levels in ME-treated mice. The proliferative functions of adenosine may be active in our model.

In brief, ME protected against AOM/DSS-induced carcinoma by reducing intestinal inflammation, repairing intestinal barrier damage, restoring gut homeostasis, and linking the microbiota and metabolites. The data above suggested that ME administration might be a possible therapeutic strategy for CAC.

## Data availability statement

The datasets presented in this study can be found in online repositories. The names of the repository/repositories and accession number(s) can be found below: NCBI - PRJNA924979.

## Ethics statement

The animal study was reviewed and approved by the Institutional Animal Ethics Committee of Nanjing University (IACUC-2003031, 18 March 2020).

## Author contributions

XT, LW, and GT: conceptualization and methodology. LT, DW, and YZ: validation and performing. XT and TW: analysis and writing. QW, ZZ, and YWe: supervision and review. FY and YWa: project administration and funding acquisition. All authors have read and agreed to the published version of the manuscript.
