# Supplementary material for: Maggot extracts chemo-prevent inflammation and tumorigenesis accompanied by changes in the intestinal microbiome and metabolome in AOM/DSS-induced mice
Source: Front Microbiol. 2023 May 2;14:1143463. doi: 10.3389/fmicb.2023.1143463 (PMC10185807; doi:10.3389/fmicb.2023.1143463)
Supplement: Supplementary file 1 [file Table_1.docx]

Supplementary Material

Maggot extracts chemo-prevent inflammation and tumorigenesis accompanied by changes in intestinal microbiome and metabolome in AOM/DSS-induced mice

Xun Tang^1,2†^, Lei Wang^5†^, Daojuan Wang^1^, Yi Zhang^3^, Tingyu Wang^1^, Zhengquan Zhu^1^, Yajing Weng^1^, Gaojian Tao^1^, Qin Wang^2^, Li Tang^2^, Feng Yan^2*^ and Yong Wang^1,4*^

^†^These authors contributed equally to this work

*** Correspondence:** [yanfeng@jszlyy.com.cn](mailto:yanfeng@jszlyy.com.cn) (F.Y.); [yongwang@nju.edu.cn](mailto:yongwang@nju.edu.cn) (Y.W.)

# Supplementary Method

**1.1. UHPLC-MS analyzing**

In both electrospray ion source (ESI) positive and negative modes, the mobile phase contained A=25 mM ammonium acetate and 25 mM ammonium hydroxide in water and B= acetonitrile. The gradient was 85% B for 1 min and was linearly reduced to 65% in 11 min, and then to 40% in 0.1 min and kept for 4 min, and then increased to 85% in 0.1 min, with a 5 min rebalancing period employed.

Information dependent acquisition (IDA) was used to acquire the product ion scan——the second mass spectrometer, which was with high sensitivity mode. The parameters were set as follows: the collision energy (CE) was fixed at 35 V with ± 15 eV; declustering potential (DP), 60 V (+) and−60 V (−); exclude isotopes within 4 Da, candidate ions to monitor per cycle: 10.

**1.2. Multivariate statistical analysis**

**1.2.1 PLS-DA**

Partial least squares discriminant analysis (PLS-DA) is a supervised dimensionality reduction method in which class memberships are coded in matrix form into Y to better distinguish the metabolomics profile of two groups by screening variables correlated to class memberships. PLS- DA was applied in comparison groups using R package ropls (<http://www.r-project.org/>).

**1.2.2 OPLS-DA**

Orthogonal projection to latent structures-discriminant analysis (OPLS-DA) is an extension of PLS-DA which incorporates an Orthogonal Signal Correction (OSC) filter into a PLS model. The basic concept in OPLS is to separate the systematic variation in X into two parts, one that is correlated to Y and one that is not correlated (orthogonal) with Y. Only the Y‐ predictive variation is used to model the data. OPLS-DA was applied in comparison groups using R package models (http://www.r-project.org/). The OPLS-DA model was further validated by cross-validation and permutation test. For cross-validation, the data was partitioned into seven subsets, where each of the subsets was then used as a validation set. R2 indicated the total variation in the data matrix that was explained by the model. Predictive ability (Q2) values represented the most recognized diagnostic statistical parameter to validate the OPLS-DA model in metabolomics. Acceptable predictive model is considered for Q2 value greater than 0.4. Good predictive model is considered for Q2 value greater than 0.9. Permutation test randomly permutes class labels for 200 times and then produces a distribution of R2’ values and Q2’ values. In essence, a reliable model should yield significantly larger R2 and Q2 value compared to R2’ and Q2’ values generated from random models using the same data set.

**1.2.3 Pathway analysis**

Kyoto Encyclopedia of Genes and Genomes (KEGG) is the major public pathway-related database that includes not only genes but metabolites. Metabolites were mapped to KEGG metabolic pathways for annotation and enrichment analysis. Pathway enrichment analysis identified significantly enriched metabolic pathways or signal transduction pathways in differential metabolites comparing with the whole background.

# Supplementary Figures and Tables

## Supplementary Tables

**2.1.1 Table S1.** The sample results of sequencing and quality control

| Sample Name | Raw Reads | Clean Reads | Raw Tags | Clean Tags | Chimera | Effective Tags | Effective Ratio (%) |
| --- | --- | --- | --- | --- | --- | --- | --- |
| NC1 | 126694 | 125942 | 108890 | 107062 | 12220 | 94842 | 74.86 |
| NC2 | 125838 | 125113 | 107542 | 105863 | 11795 | 94068 | 74.75 |
| NC3 | 125613 | 124854 | 107199 | 105187 | 11133 | 94054 | 74.88 |
| NC4 | 128607 | 127917 | 112333 | 110855 | 12866 | 97989 | 76.19 |
| NC5 | 126567 | 125794 | 108619 | 106763 | 11562 | 95201 | 75.22 |
| A_D1 | 118398 | 117911 | 106743 | 105728 | 10768 | 94960 | 80.2 |
| A_D2 | 128852 | 128209 | 114122 | 112772 | 10791 | 101981 | 79.15 |
| A_D3 | 49840 | 49681 | 44438 | 43859 | 3603 | 40256 | 80.77 |
| A_D4 | 123936 | 123363 | 110869 | 109720 | 11310 | 98410 | 79.4 |
| A_D5 | 129948 | 129374 | 111839 | 109487 | 10611 | 98876 | 76.09 |
| A_D_ME1 | 69284 | 69000 | 62439 | 61781 | 6964 | 54817 | 79.12 |
| A_D_ME2 | 135616 | 134998 | 121023 | 119518 | 14407 | 105111 | 77.51 |
| A_D_ME3 | 135198 | 134540 | 119894 | 118414 | 14367 | 104047 | 76.96 |
| A_D_ME4 | 137028 | 136402 | 121904 | 120460 | 14154 | 106306 | 77.58 |
| A_D_ME5 | 127761 | 127243 | 114714 | 113438 | 13558 | 99880 | 78.18 |
| ME1 | 131311 | 130753 | 113945 | 112248 | 11858 | 100390 | 76.45 |
| ME2 | 133820 | 133278 | 115842 | 114031 | 12047 | 101984 | 76.21 |
| ME3 | 133572 | 132911 | 116964 | 115356 | 12574 | 102782 | 76.95 |
| ME4 | 126702 | 126190 | 111402 | 109872 | 12510 | 97362 | 76.84 |
| ME5 | 131939 | 131357 | 115801 | 114141 | 11857 | 102284 | 77.52 |

**2.1.2 Table S2**. The alpha diversity analysis of different samples

| Sample Name | Shannon | Simpson | Chao | Goods’_coverage | Pielou | pd |
| --- | --- | --- | --- | --- | --- | --- |
| NC | 5.63±0.24 | 0.95±0.01 | 840.42±39.77 | 1.00±0.00 | 0.59±0.02 | 100.17453 |
| A_D | 4.23±0.44 | 0.83±0.04 | 763.29±311.69 | 1.00±0.00 | 0.45±0.02 | 96.05652 |
| A_D_ME | 5.00±0.30 | 0.91±0.03 | 803.43±95.87 | 1.00±0.00 | 0.53±0.02 | 93.427724 |
| ME | 4.95±0.18 | 0.89±0.02 | 1173.10±4.81 | 1.00±0.00 | 0.49±0.02 | 165.316458 |

## Supplementary Figures

**Supplementary Figure 1.** ME demonstrate no toxicity in mice with ME administration. (A)ALT, AST, CRE and URE levels in the serum;(B) liver, kidney, spleen and lung were sectioned and stained with H&E. Scale bar=200 µm

**Supplementary Figure 2.** overall view of colon wrapped into the “Swiss-rolls”. solid line, adenocarcinoma; dotted line, dysplasia. (A: the A_D group, B: the A_D_ME group)

**Supplementary Figure 3.** the proportion of abundance at phylum level in negative control (NC) and CAC modal(A_D) treated with me (A_D_ME and ME) or not
