# Supplementary figures and images for: Maggot extracts chemo-prevent inflammation and tumorigenesis accompanied by changes in the intestinal microbiome and metabolome in AOM/DSS-induced mice
Source: Front Microbiol. 2023 May 2;14:1143463. doi: 10.3389/fmicb.2023.1143463 (PMC10185807; doi:10.3389/fmicb.2023.1143463)

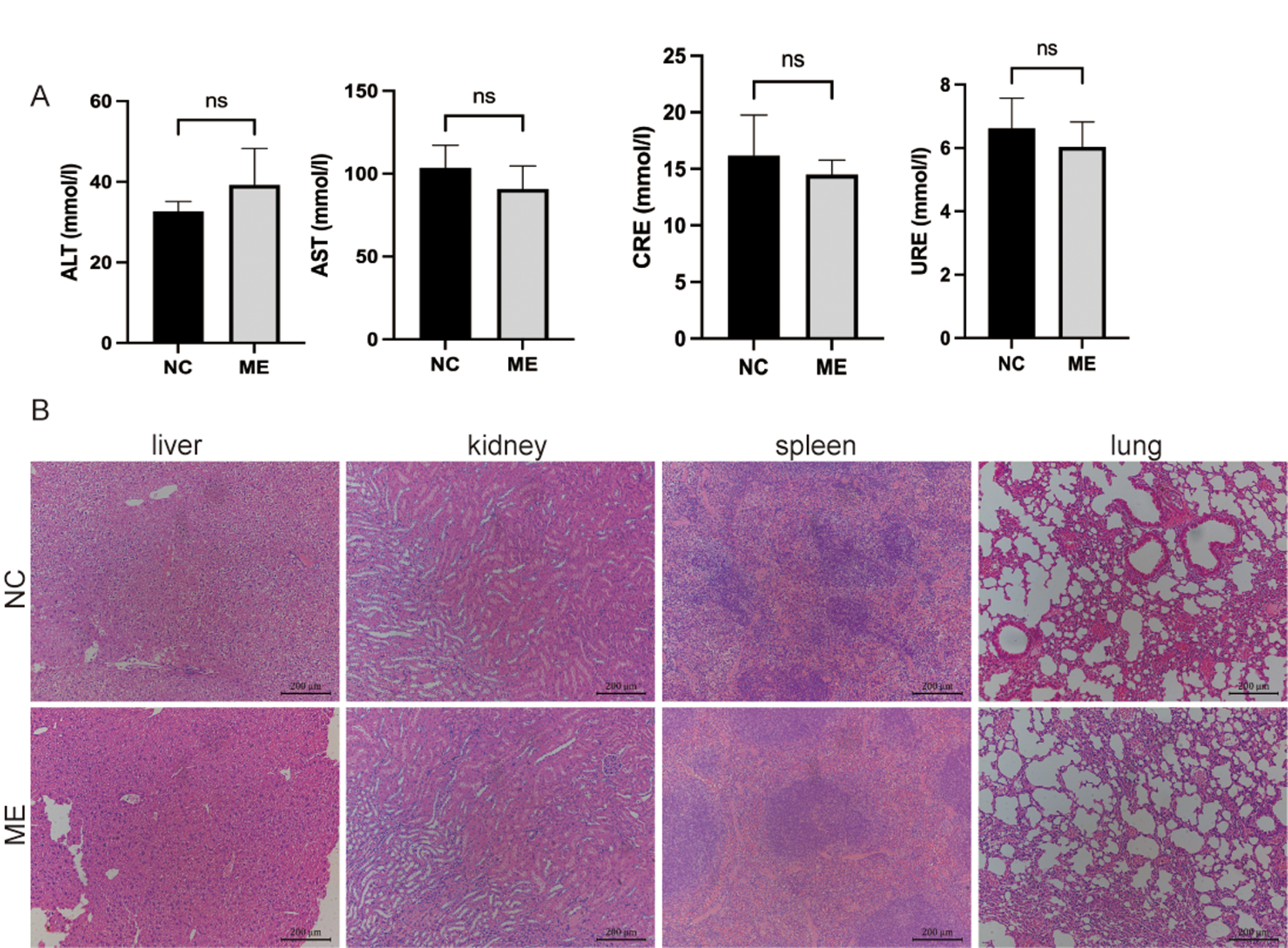

Supplement: Supplementary file 2 [file Image_1.tif]

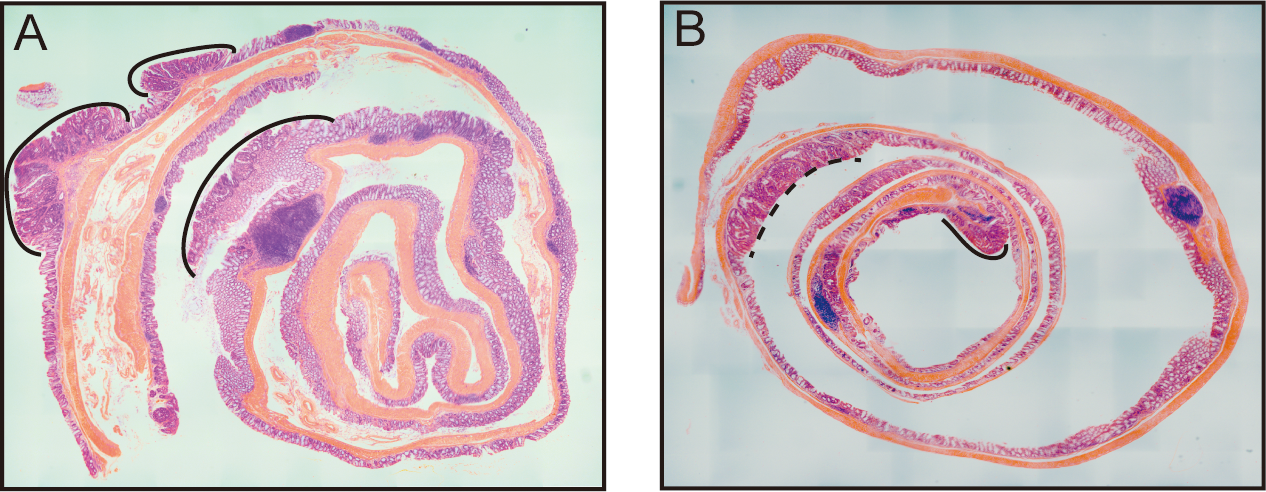

Supplement: Supplementary file 3 [file Image_2.tif]

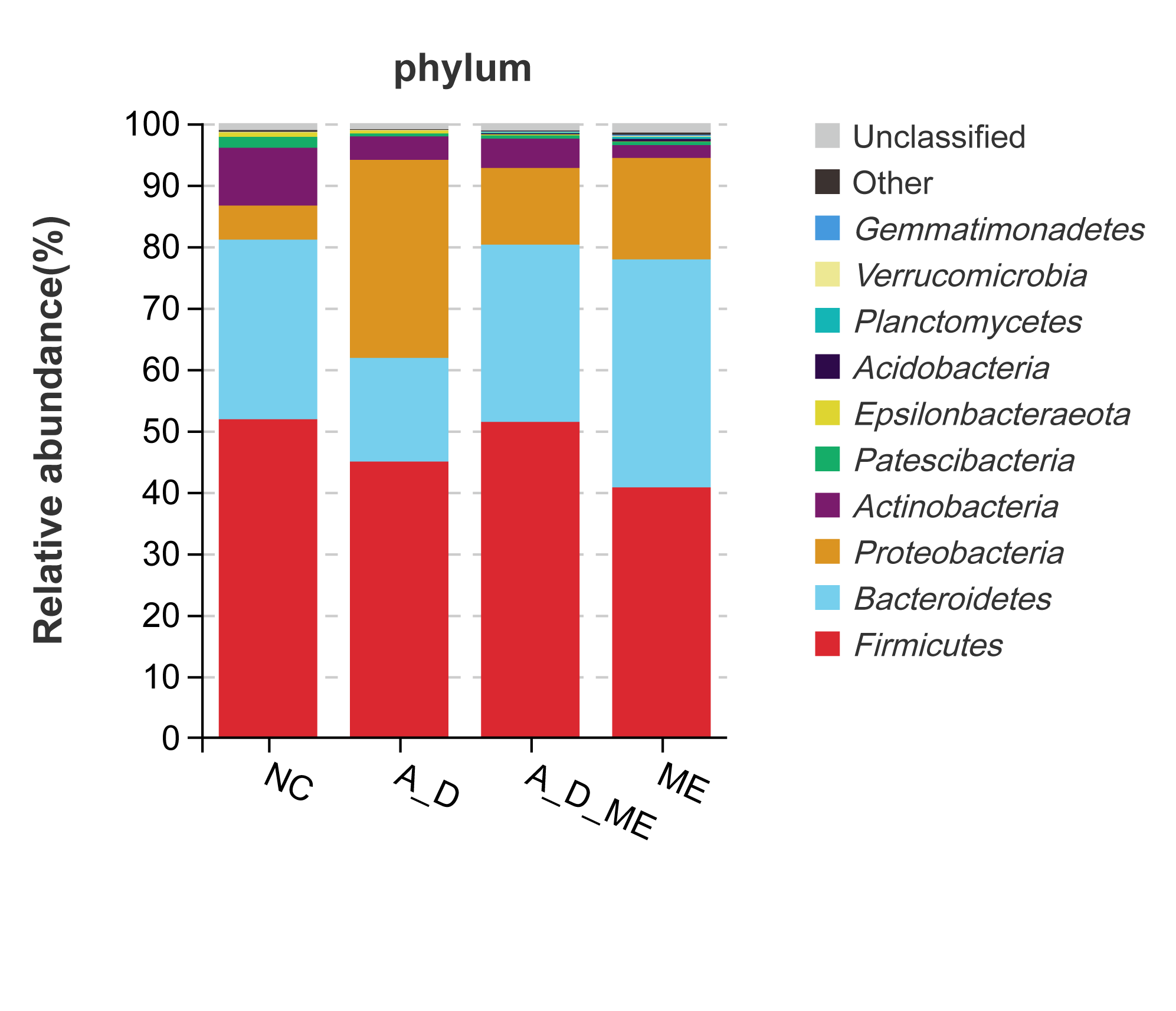

Supplement: Supplementary file 4 [file Image_3.tif]
